# Noises on—How the Brain Deals with Acoustic Noise

**DOI:** 10.3390/biology13070501

**Published:** 2024-07-04

**Authors:** Livia de Hoz, David McAlpine

**Affiliations:** 1 Neuroscience Research Center, Charité—Universitätsmedizin Berlin, 10117 Berlin, Germany; 2 Bernstein Center for Computational Neuroscience, 10115 Berlin, Germany; 3 Department of Linguistics, Macquarie University Hearing, Australian Hearing Hub, Sydney, NSW 2109, Australia

**Keywords:** auditory, noise, background, foreground, statistical learning, feedback, loops, inferior colliculus, auditory cortex

## Abstract

**Simple Summary:**

The listening brain must resolve the mix of sounds that reaches our ears into events, sources, and meanings. In this process, noise—sound that interferes with our ability to detect or understand sounds we need or wish to—is the primary challenge when listening. Importantly, noise to one person, or in one moment, might be an important sound to another, or in the next. Despite the many challenges posed by noise, however, human listeners generally outperform even the most sophisticated listening technologies in noisy listening environments. Because extracting noise from the sound stream is a fundamental process in listening, understanding how the brain deals with noise, in its many facets, is essential to understanding listening itself. Here, we explore what it is that the brain treats as noise and how it is processed. We conclude that the brain has multiple mechanisms for detecting and filtering out noise, and that incorporating cortico-subcortical ‘listening loops’ into our studies is essential to understanding this early segregation between noise and signal streams.

**Abstract:**

What is noise? When does a sound form part of the acoustic background and when might it come to our attention as part of the foreground? Our brain seems to filter out irrelevant sounds in a seemingly effortless process, but how this is achieved remains opaque and, to date, unparalleled by any algorithm. In this review, we discuss how noise can be both background and foreground, depending on what a listener/brain is trying to achieve. We do so by addressing questions concerning the brain’s potential bias to interpret certain sounds as part of the background, the extent to which the interpretation of sounds depends on the context in which they are heard, as well as their ethological relevance, task-dependence, and a listener’s overall mental state. We explore these questions with specific regard to the implicit, or statistical, learning of sounds and the role of feedback loops between cortical and subcortical auditory structures.

## 1. The Challenge of Noise

*‘Nothing essential happens in the absence of noise.’*

(Jacques Attali, French economist and philosopher)

Noise—which we define here as interfering sounds that mask what we are trying to hear or that divert our attention—is the primary challenge in listening. The listening brain must resolve the mix of sounds that reaches our ears into events, sources, and meanings that unfold over multiple cadences. Making sense of sound relies on separating what we want to hear—‘signals’—from what we do not—‘noise’ [1]. Importantly, noise to one person, or in one moment, may be signal to another, or in the next. Processing noise (filtering it out or using its potential predictive power) is something the brain achieves seemingly effortlessly, but it is not obvious how. Normal-hearing listeners are adept at ‘cocktail-party listening’ despite the often very high levels of background noise, yet the cognitive effort involved in this process is revealed in hearing-impaired listeners, who struggle to make sense of sound in even moderately challenging environments. Despite the many challenges noise presents, humans generally outperform even the most sophisticated listening technologies and are much better at extracting and parsing information and meaning from sounds that add context and ‘texture’ to listening.

And yet, we know little about what is classified as noise by the brain, and when so, and how and why the neural representation of noise is removed (the process of ‘denoising’) in the brain. Some sounds are always, or mostly, noise and rapidly relegated to ‘background’ (e.g., a waterfall, strong wind, a sudden downpour). Other sounds are, or become, ‘background’ (noise) when we wish to attend to ‘foreground’ sounds; a talker in a crowded room mentioning our name, perhaps. Loud or quiet, continuous or sporadic, embedded in the signal or generated by an entirely different source, the intensity, and texture of noise, all have a strong influence on how it is interpreted.

In this review, we address the question ‘what is noise?’ from perceptual and biological perspectives. We focus on those aspects of sound processing that enable us to determine what the auditory brain might at least consider noise. We discuss the effects of masking noise on perception and describe phenomena to illustrate how the brain has evolved to deal with noisy listening environments, including through binaural hearing. We then discuss how the brain adapts to background noise and how noise-invariant representations of sound might arise. Finally, we discuss how noise seems to automatically be incorporated into our perception of the world, and how it triggers sensory and motor actions to overcome its impact on our communication abilities. Two themes recur: that the brain contains mechanisms for detecting and dealing with noise implicitly—i.e., without engagement—and that subcortical structures are key to this process.

## 2. Noise as a Source of Interference—Energetic and Informational Masking

It is usually the case that noise of sufficient intensity harms listening performance by masking the signal we ought to hear (a predator approaching against strong wind in a forest). Indeed, sound intensity is a key determinant of how effective one sound is in masking another, with a winner-takes-all effect by which the energy of the signal determines its competitive strength, especially in the auditory periphery. Release from this ‘energetic’ masking can arise when the signal and the noise originate from different locations, a phenomenon known as spatial release from masking [2], and facilitated by binaural hearing (discussed below) or when the two signals hold different spectral compositions; for example, in terms of a talker’s voice release [3]. When the masking sound is modulated, listening to the signal in the modulation minima—the trough of the noise signal or ‘dip-listening’—can occur, and it is possible to follow a conversation if sufficient snippets of what a talker is saying are secured [4,5]. Nevertheless, even without dip-listening, the independence (from each other) of auditory channels provides a significant boost to following a conversation in external noise [6]. Perhaps not surprisingly, the specific nature of noise determines its masking capacity. Broadband noise, for example, despite its wide spectrum, is less detrimental to speech understanding compared to other forms of spectrally selective masking. Recent evidence suggests that the specific form of masking noise invokes very different brainstem, midbrain, and cortical circuits, even for the same level of speech understanding [7]. When listeners are engaged in the task of detecting non-words in a string of noisy words, vocoded speech—an intrinsically noisy representation—invokes brainstem circuits through the medial olivo-cochlear (MOC) reflex to sharpen cochlear filtering, supporting listening performance. For the same level of performance in the task, speech-shaped noise (noise with the same long-term spectrum of speech) elicited elevated levels of brainstem and midbrain auditory activity, whilst multi-talker babble—a particularly challenging form of masker—elicited the greatest level of cortical activity, including processing indicative of greater cognitive engagement. These differences were less evident, or altogether absent, during passive listening, suggesting that the listening brain is equipped with multiple mechanisms—from ear to cortex and back—for dealing with different forms of noise and different mental states.

### Informational Masking

Beyond energetic masking, the concept of ‘informational’ masking has been used to describe situations in which non-overlapping sound energy nevertheless impairs (masks) speech understanding (see review in [8]). Intuitively, this makes sense; hearing a conversation against a background of other talkers, for example, likely requires active engagement in listening and the use of cognitive resources. Even when the acoustic energy of two talkers is largely non-overlapping, information conveyed by the unattended talker can impact that conveyed by the attended; it becomes noise for the purposes of understanding. Nevertheless, defining noise as energetic (overlapping) or informational (non-overlapping) is perhaps a simplification. Neural activity generated by any sound, even in the auditory periphery brainstem, is not instantaneous. For example, evidence that a time-reversed speech signal generates less informational masking than forward-running speech [9] assumes that masking relates to semantic meaning—reversed speech (noise) has the same energy as the signal (forward speech) but, being unintelligible, is less effective as an informational masker. However, natural speech also contains a very different temporal waveform to reversed speech—a series of rapid acoustic attacks—favored by biophysical mechanisms responsible for neural firing—and slower decays, whilst the temporal waveform of reverse speech shows the opposite pattern—slow rises in energy and rapid decays—likely eliciting fewer and less-precise neural responses [10]. Beyond acoustics, but well before semantics, forward and reverse speech elicit different amounts of ‘neural’ energy. *In extremis*, non-overlapping acoustic transients (clicks) can be rendered temporally inseparable by the ‘ringing’ response of the basilar membrane (see the discussion of the precedence effect, below), rendering their overlapping status moot. Whilst energetic and informational masking are useful concepts in describing acoustics and perception, their neural counterparts are currently less well-conceptualized.

## 3. Binaural Hearing—The Brain’s Denoising Algorithm

Though many factors likely contribute to successful listening in noise, including a healthy inner ear, it is indisputably the case that binaural, or two-eared, hearing is key to successful ‘cocktail-party listening’, the ability to follow a conversation against a background of noise, competing voices, or reverberant sound energy [11]. Possessing two functional ears provides us with an enormous listening benefit beyond the 3-decibel benefit provided by two independent sound receivers or even the ability to locate sources of sound based on sensitivity to binaural cues—interaural time and level differences (ITDs and ILDs, respectively).

**Figure 1:**
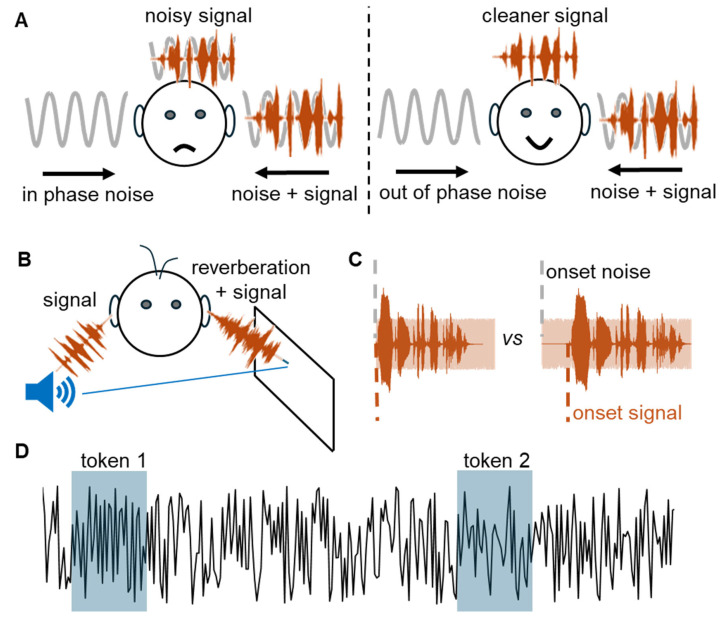
Schematics of noise effects in different contexts. (**A**) Binaural unmasking as a result of out-of-phase binaural noise. On the left, the noise coming into the left and right ears is in phase such that the signal (copper) coming into the left ear is only partially denoised by the brain, as shown by drawing above the head. On the right, the noise coming into the left and right ears is out of phase and the signal (copper) is perceived more cleanly by the brain, as shown by the drawing above the head. Inspired by [15]. (**B**) Hearing in reverberating environments. The sound (copper) arrives directly from the source (blue speaker) into the right ear and indirectly, as a reflection from the wall and contaminated by reverberation, into the left ear. (**C**) The advantage of pre-exposure to background sound. On the left, the vocalization (copper wave) and the background noise (pink area behind) start at the same time. On the right, the vocalization starts later, a condition that facilitates its understanding. (**D**) Textures vs. exemplars. The specific statistics of two short tokens of sound (1 and 2) can differ from one another (token 1 contains higher frequencies than token 2) and from the summary statistics of a long texture sound.

The most well-known and well-studied of the binaural benefits is binaural unmasking (see [12] for a comprehensive review), an improvement in the detection of sounds or the intelligibility of speech in background noise based on the relative interaural configurations of the signal and masker (noise). Independently reported in 1948 [13,14], the ability to hear out sounds in background noise by inverting the fine-structure phase of the low-frequency signal (<1500 Hz) or masker waveform at one ear relative to the other has spawned an entire research field and supports a wide range of audio technologies, listening devices, and therapeutic interventions (Figure 1A). The remarkable denoising capacity of binaural hearing is demonstrated most compellingly in that it overrides the addition of sound energy *per se* to accrue a listening benefit. Specifically, if a tone and a fixed level of masking noise are presented monaurally to the same ear and the level of the tone reduced to the point it becomes undetectable, the tone becomes detectable again simply by adding identical noise to the other ear. Its level must be reduced once more to reach the ‘masked threshold’. Remarkably, adding an identical noise to the other ear makes it easier once more to hear the signal. Then, by adding an identical tone to the other ear—such that both signal and noise are identical across the ears—the tone becomes more difficult to detect, despite the overall increase in signal energy across the ears—necessitating an increase in level to perceive it. Finally, by inverting the phase of the tone or the noise in one ear relative to the other, the tone becomes audible again, more so when the tone is inverted—the classic binaural unmasking paradigm (Figure 1A). This counterintuitive effect relies on brain mechanisms that compare the relative phases of the signal and the masker at each ear, and the relative difference in signal and masker phase across the ears determines the magnitude of the unmasking benefit. Clearly evolved to support real-world listening in noisy environments, binaural unmasking combines with the effect of head shadowing and perhaps even the absolute sensitivity to monaural sound levels to allow spatial release from masking—the ability to hear sounds based on the relative location of specific sources and interfering noise.

### Neural Mechanisms for Binaural Unmasking

A neural basis for binaural unmasking was established in a series of in vivo experiments confirming the existence of neurons in the midbrain—likely innervated by inputs from primary binaural brainstem neurons—that demonstrate features consistent with the perceptual effect and existing models [16,17,18]. Specifically, neurons in the inferior colliculus sensitive to ITDs show lower thresholds to out-of-phase binaural tones than to in-phase binaural tones in binaural background noise [18]. Brain mechanisms contributing to this ability are likely highly conserved across species, with an exception being fish [19]. To this point, a series of experiments across species, brain centers, and recording modalities by Dietz and colleagues demonstrated that the auditory brain’s capacity to deal with more realistic types of sound in reverberant environments relies on fast temporal capacity of binaural processing, facilitating the extraction of reliable spatial cues arriving direct from the source and suppressing before the later arrival of more- energetic, reverberant sound energy in which spatial cues are scrambled [20,21]. Importantly, this binaural form of denoising relies on monaural inputs that are already strongly adapted at the level of the auditory nerve; modeling data [22] suggest that human speech sounds in reverberation are consistently mis-localized in the absence of monaurally adapting inputs to binaural neurons. Brain mechanisms supporting binaural hearing seem highly evolved to cope with noisy and reverberant environments, with access to temporal information conveyed in low-frequency sounds particularly important in achieving this.

## 4. Listening Spaces as a Source of Noise—Dealing with Reverberation

One common source of noise we often must deal with is reverberant sound energy, or echoes (Figure 1B). Most natural, including open, spaces generate acoustic reverberations [23], and these can provide useful information as to the size, nature, purpose and importance of the spaces we inhabit [24]. Often, the direct-to-reverberant ratio (DRR) can be negative, i.e., sound energy direct from the source can be less intense than later-arriving reflections and interference patterns, yet normal-hearing listeners seem able to parse these complex environments with relative ease. Even when absolute listening performance is impaired by reverberation, our brains have evolved to deal with it to some degree. For example, listeners seem able to adapt to the reverberant characteristics of rooms [25] with just a few milliseconds of prior listening exposure to the sounds in a room supporting better speech understanding (Figure 1C) [26]. This benefit does not accrue in anechoic environments, suggesting that parsing speech in noisy, reverberant environments taps into brain mechanisms that implicitly learn the acoustic features of background sounds to improve explicit processing of foreground ones. Rather than the now common act of applying denoising algorithms to remove (presumed) external noise though listening devices (‘hearables’, earbuds, and the like), listeners might benefit from being pre-adapted to noisy and reverberant listening environments in which they need or wish to communicate [1]. Research fields such as eco-acoustics have developed in acknowledgement of the fact that listening is optimized for real-world spaces. This becomes more important as the world becomes noisier, with initiatives taking place to try to preserve natural soundscapes, for example, the ‘One square inch of silence’ [27]. Individual listeners are often ignored in our listening spaces, with other sources of ‘noise’—cognition, hearing status, age, language, and culture—rarely featuring in their design, construction, or assessment.

### The Precedence Effect—Direct Is Correct

Given our need to navigate the spaces we inhabit, reverberation also generates ambiguities in terms of the perceived location of sound sources. In particular, despite the presence of multiple reflected copies of the sound arriving from different locations milliseconds later, listeners usually report a single sound image originating from its source. This dominance of directional cues conveyed by earlier-arriving information has been explored through a set of phenomena collectively referred to as the precedence effect (sometimes referred to as the ‘Haas’ effect [28]). In one common paradigm used to explore precedence, two identical sounds are presented from two different speakers, whether simultaneously or with a delay below the so-called echo threshold, so they will be heard as a single sound (‘fusion’). As the delay is increased further, the leading sound dominates the perceived location until, for delays that are longer still, two, spatially distinct, sources are perceived. Together, localization dominance and fusion permit more accurate processing of sources in acoustically reverberant environments [29]. In vivo physiological studies of the precedence effect report reduced neural responses to lagging sounds not explicable purely by neural adaptation [30,31]. Neural correlates have been suggested at the level of the inferior colliculus [32] (see also ref. [33]), in which neural responses to lag stimuli are suppressed up to 40 ms after the presentation of a lead stimulus [34]. The magnitude and time course of neural responses to lagging clicks, which depend on the ITDs imposed on leading and lagging clicks, have been correlated with behavioral measures of precedence [30,31,33], suggesting an ITD-dependent inhibition that elicits a temporary break in interaural sensitivity [35]. This neural-suppression hypothesis assumes neural responses to each binaural click are processed separately and in sequence. Despite widespread acceptance of this view, however, an alternate hypothesis suggests the psychophysical effect is explicable without recourse to neural inhibition or, indeed, any central auditory processing. Instead, the finite response time of the basilar membrane to transient stimuli, especially at the apical, low-frequency end of the cochlea, renders leading and lagging sounds a single event on the basilar membrane [36,37]. Depending on the interval between them, the amplitude and timing cues conveyed by leading and lagging clicks will be modified such that, following binaural integration, the internal representation of directional cues differs from that presumed from the external stimuli. For species that exploit directional cues present in the low-frequency components of sound, localization judgments would be determined by these altered cues.

Regardless of the precise mechanisms contributing to precedence, the ‘echo-threshold’ can vary substantially depending not only on the type of sound (up to about 5 ms for clicks and up to 40 ms for speech or other complex sounds) but also on the environment. For example, in anechoic and reverberant environments, sounds arriving from opposite sides will be fused for delays of up to about 5 and over 30 ms, respectively [38,39]. Broadband noise superimposed over this time interval will have little effect on these thresholds, again supporting the notion that broadband noise, unless overly intense, is easily filtered out by the brain.

Overall, whether through mechanical or computational means, the logic of physics prevails—sound arriving direct from a source is likely to arrive earlier than its echo(es) and the brain ‘accepts’ this implicitly.

## 5. Filling in the Gaps—Noise as a Masker Even When It Is Not

Although our brains evolved in natural soundscapes and environments, synthetic and illusory sounds can also be informative as to the brain’s function and its implicit sensitivities. This is particularly true of illusions that result in the preservation (or creation) of stable percepts. One such illusion of relevance to how the brain deals with noise is the ‘continuity illusion’. Tones presented as interrupted pulses are perceived as such until the silent gaps in between are filled with noise. This simple manipulation transforms the sound percept from one of disconnected tones to one of a single, continuous tone pulse. This illusion is stable across species, including humans [40,41,42,43,44], non-human primates [45,46,47] and cats [48]. It is also resilient to the type of sound, from modulated tones [41,42,49], music [50], and vowels [51], to more complex speech stimuli [52]. Interestingly, the illusion only occurs when the noise fills the gap, not when the noise is presented continuously over both the gap and sounds [40,42,44,45]. While the mechanisms are not understood, neurons in the primary auditory cortex of cats and monkeys respond to the illusion as if it was a continuous sound, but not to the individual tones, nor to the noise alone [46,48]. Once more, broadband noise is automatically blended into the background.

## 6. Responding Reflexively to Noise—The Lombard Effect

The brain’s adaptations to communication in noise extend beyond those that enhance the signal-to-noise ratio of what we hear to increase the intensity of what we emit. When background sound intensities increase, animals respond by automatically and involuntarily vocalizing more intensely. In humans, this ‘Lombard reflex’ (named for its discoverer) extends to speech properties such as the rate and duration of syllables [53]. Whilst the Lombard reflex evolved to be dynamic in environments with fluctuating intensities, the overall noisier urban spaces of the 21st century affect how we and other animals vocalize. This became evident during the COVID-19 pandemic, for example, when birds in the San Francisco Bay area reduced the intensity of their song, presumably as a response to the measurable reduction in the intensity (and occurrence) of environmental sounds during the period of enforced lockdown [54].

The Lombard reflex has been observed in numerous vertebrate species from fish to birds and mammals, with few exceptions [55], and thus has a possible evolutionary origin dating as far back as 450 million years. While the mechanisms remain unknown, the fact that it is evident in decerebrate cats and fish supports the importance of subcortical structures in its emergence. Its rapid latency relative to intensity increases also supports a role for subcortical circuits. While latencies of ~150 ms have been measured in humans and other animals [56,57,58], in bats the increase in the call amplitude can occur as fast as 30 ms after sound onset [59], too soon for higher-order brain structures to be involved or feedback on the intensity of self-vocalizations to be assessed. The effect tracks not only upward but also downward changes in the background intensity, so long as these occur over time windows of >50 ms in bats [59], suggesting temporal summation on time scales of tens of milliseconds. Two lines of evidence suggest that cortical influences support the emergence and magnitude of the effect. While the Lombard reflex is normally automatic, subjects can learn to control it using, for example, visual feedback on the loudness of their voices [60]. Another clue to the circuit mechanisms can be derived from the frequency-specificity of the reflex. While most studies use broadband noise to induce the reflex, using narrower band noise has revealed, in birds, bats, humans and monkeys, that increases in amplitude only occur when the noise covers the spectral band of the vocalization [61,62,63,64]. Thus, the circuit shows some precision in its tonotopic specificity. Whether this is learned or hard-wired is not known.

## 7. Stochastic Noise and the Brain’s Internal State

It is worth noting that some noise is internal, and not all of it is bad. Within the inner ear, ‘stochastic’ noise—random fluctuations in input—such as Brownian motion in the mechano-transducing sensory hair cells, far from harming performance, improves their sensitivity, whilst the high (often >100 s^−1^) spontaneous firing rates of auditory nerve fibers render them independent of each other, enhancing the flow of information to the listening brain [65]. Stochastic noise might even expand the information bottleneck in cochlear implants [66] and could inform new signal-processing strategies that convey a richer listening experience in listening devices [67].

Despite the apparent utility, however, elevated levels of internal noise are generally considered a form of pathology, with maybe the most relevant of these being tinnitus—the perception of sound in the absence of any external sound source. The phenomenon of tinnitus illustrates the delicate balance in the adult brain between external input and internal activity and how a disconnection between the two might have the effect of generating unwanted internal noise in the system beyond a mere reduction in sensitivity. Affecting maybe 10% of the population, and afflicting half of these, tinnitus (or ’ringing in the ears’) is long associated with damage to the sensory hair cells responsible for our sensitive hearing [68]. So-called *objective* tinnitus can be understood as the result of compensatory, homeostatic upregulation of neural activity (or down-regulation of neural inhibition) in the central nervous system, perhaps to maintain some required long-term level of neural responsiveness. This maladapted neural gain generates elevated neural ‘noise’—neural firing not related to external stimulus—with the concomitant perceptual experience of sound. This explanation once fell short when trying to account for *subjective* tinnitus, tinnitus that arises without any obvious sign of inner ear or brain pathology. Recently, however, the concepts of cochlear synaptopathy [69]—pathologies to synaptic transmission in a specific population of high-threshold inner-ear nerve fibers that code for higher sound intensities—suggests an occult form of hearing deficit (coining the term ‘hidden hearing loss’ [70]) that manifests as tinnitus as well as problems listening in background noise (‘I can hear you but I can’t understand you’) in otherwise normal-hearing listeners. This perspective is supported by in vivo research demonstrating that neural coding of speech in background noise [71] and the ability to adapt to different noisy environments [72] are both impaired by synaptopathic insults to the inner ear that spare hearing thresholds. A recent theoretical report suggests that feedforward adaptive changes in the level of internal stochastic noise arising from hearing loss—including ‘hidden’—can explain phantom percepts such as tinnitus in the context of the Bayesian brain framework [73]. In this perspective, a mismatch between the brain’s *a priori* expectations and the input results in neural noise being interpreted as signal. An important facet of this construct is the brain’s expectation of a familiarized level of internal, stochastic noise along the auditory pathway, and a resulting pathology of a phantom percept when the level of internal noise changes.

## 8. From Foreground to Background—And Back

Whether foreground or background, signal or noise, all the sounds processed by the inner ear likely impact the auditory brain, a key distinction being the extent to which they are consciously experienced (foreground more than background). Given the great many sounds that might impinge on us at any one time, there is a clear advantage to processing many of these subconsciously; background sounds representing a form of ‘aural wallpaper’ to be accessed more fully when it becomes important to do so. A relatively recent perspective on the issue of how this is achieved takes a more ethological approach than traditional psychoacoustics in terms of considering how the listening brain deals with the complexities of cluttered acoustic scenes—the classic Bregmanian ‘auditory scene analysis’ problem [40,44]. These studies are often predicated on the fact that (1) whilst foreground sounds must be attended to, background sounds—‘noise’—must also be processed in order to provide access to the entire auditory scene, including to sounds that might soon become foreground or that represent a lurking danger, perhaps, and (2) that the brain has evolved specifically to deal with this problem, particularly over time. Lines of investigation explore how the auditory system detects order (signal) from disorder (noise)—evidence, perhaps, of a source emerging from the background. Employing sequences of tones that transition between random and regular patterns, these studies demonstrate a clear difference in processing time and the associated neural signatures depending upon whether a transition is from random to regular or vice versa [74]. Specifically, determining that a sequence contains some form of acoustic structure takes longer to process than departures from regularity once knowledge of regularity is established [75], and this process is more affected by interfering acoustic events than visual ones [76]. Another way of exploring this issue concerns the extent to which ‘textures’—naturalistic background sounds such as fire, water, wind, rain etc.—are represented in the brain not as objects *per se* but in terms of their summary statistics, at least for sounds of sufficient duration [77]. Again, there is a clear temporal dimension to this type of processing; short ‘tokens’ of sound textures are perceived as objects, distinguishable from other tokens drawn from the same statistical pool. With increasing duration, however, individual tokens of textures are less distinctive from each other, and eventually indistinguishable, whilst sensitivity to their longer-term ‘summary’ statistical structure increases. This suggests a continuum over which foreground and background sounds are not distinct, but rather are processed as if they exist along the same temporal and statistical dimensions. Sound objects emerge from noise, or fade into it, depending on their positions along these dimensions.

However, although the ability to process background sounds absent obvious conscious engagement is likely of evolutionary advantage, a potential confounder in these studies is the extent to which listeners can suppress seemingly innate abilities to impose some form of structure on them or meaning to them other than the stimulus dimension presumed in the experimental paradigm. Specifically, though expressed in terms of the automatic, potentially subconscious, neural processing of sounds, these studies, by requiring subjects to make explicit distinctions between noise bursts [78], sound textures [77], or streams of tones [74], ‘force’ supposedly background sounds to the foreground. Furthermore, for noise bursts and sound textures at least, casting listening tasks in terms of perceptual sensitivity to summary statistics can require the removal of most (up to 95%) of exemplars on the basis that they are insufficiently ‘statistical’ in nature (i.e., they contain potential sound features to which listeners might be sensitive). If abstract or natural sounds cannot simply be condensed to their summary statistics, perhaps the main task of the listening brain is constantly to suppress the perception of spurious events and sound objects rather than straining to extract them in the first place.

### Dichotic Pitches—Creating Sounds Objects from Noise

Given the propensity of human listeners to generate the percept of sound objects from seemingly unstructured and random sounds, including noise (auditory pareidolia, often considered in terms of hallucinations; [79]), it is perhaps unsurprising that trying to account for performance in listening tasks directed toward supposedly background sounds (i.e., turning the background to foreground) remains fraught. To this end, it is worth asking what minimum sound features are required to generate a percept of foreground sounds (objects) against a background of noise. Though seldom expressed this way, the concept of dichotic pitches is telling. Dichotic pitches are clear percepts of sound objects generated when otherwise interaurally incoherent noise (independent samples of noise presented to either ear simultaneously) is briefly transitioned to coherent by applying deterministic phases and magnitudes to the signal (either the whole signal or at specific frequencies, [80]). Like sound textures, very short snippets of incoherent noise are perceived as punctate sound objects holding a distinct intracranial location determined by their combination of interaural time and level disparities. This punctate, and localizable, percept is lost, however, as the stimulus duration is increased, to be replaced by a relatively broad intracranial percept of inchoate noise [81,82]. Applied in different frequency bands over time, transitions from incoherent to coherent noise are sufficiently robust to generate melodies, distinguishable against an otherwise indistinct, noisy background [83]. This is despite the phase and magnitude transitions being imperceptible at either ear alone. Based on our knowledge of binaural hearing, the neural signature for dichotic pitches likely arises first post-synaptically in the superior olive, three synaptic stages beyond the inner ear, demonstrating the listening brain’s capacity to extract features from background noise even in the absence of any externally imposed modulation in sound energy.

## 9. Brain Circuits and Listening Loops

In seeking to understand how noise is represented in the brain, one common approach has been to study the extent to which the neural representation is invariant to noise. Such a representation, which would favor a neural representation of the signal, would be evidence for a brain mechanism of background suppression. Typically, a foreground signal, speech for example, is presented in a background of noise of different intensities, generating varying signal-to-noise ratios (SNRs). The study of noise-invariant sparse representations has focused on cortical structures and indeed neurons across sensory modalities [84] and, within the auditory modality, across species [85,86,87,88,89], have been found to show noise-invariance. Cortical noise-invariant representations are not incompatible with representations of noise itself in the same structures. For example, for noises of sufficient intensity, a substantial fraction of neurons in the cortex of the cat responded to a bird chirp embedded in noise in the same way they would to the noise alone, despite the chirp being considerably louder than the noise [90]. These data reiterate the question of ‘when is noise noise and when is it signal?’, and raises the issue of acoustic saliency. Does background noise, when sufficiently intense, impose itself to become part of the foreground? These studies often use speech or vocalizations as the signal embedded in naturalistic noises. It is possible that the use of a high-level signal anchors the invariance to higher cortical regions where speech is encoded. In any case, it is not unreasonable to think that for a noise-invariant representation to arise, the detection and subtraction of noise-evoked or -associated neural responses might have taken place upstream in the pathway. Evidence of just this has been demonstrated at the level of the cochlear nucleus in mammals [91] and the avian midbrain [92]. That noise might gradually be extracted from the signal along the auditory hierarchy is supported by electrophysiological studies [88] reporting increased noise invariance between the inferior colliculus and the auditory cortex, and from theoretical considerations [93]. Nevertheless, some across-hierarchy comparative studies report that the brain centers showing the most noise-invariant representation are the auditory thalamus and inferior colliculus [94,95] rather than cortex. In an anesthetized guinea pig, the presence of noise reduces neural discrimination of vocalization tokens across all the structures from the cochlear nucleus to the primary auditory cortex, probably because of the attenuation of slow amplitude-modulated cues that provide the temporal envelope for the test vocalizations. Interestingly, the inferior colliculus and, to a lesser extent, the thalamus provided the best discrimination level in masking noise [95]. The authors extended these results to vocalization presented in stationary or chorus (colony of vocalizing guinea pigs) noise and again the inferior colliculus and thalamus represented vocalizations with the highest fidelity [94], while neurons in the cochlear nucleus displayed the most faithful representation of the noise itself. Increasingly, the cortex is explored in terms of it being a source of feedback instruction to subcortical centers and, in this regard, inactivation of cortex bilaterally (through cooling) in an anaesthetized guinea pig abolishes the extent to which neurons in the inferior colliculus ‘learn’ over time the statistical distribution of the intensities of noise to speed up their adaptive capacity [96].

Nevertheless, it is not a question of whether auditory neurons respond to tokens of noise but of at what level of processing is the presence of background noise detected and the response to it extracted, especially when it forms a continuous background (energetically) masking potentially relevant signals. Context plays a crucial role in the interpretation of noise. For example, whether we interpret an intense background noise as an innocuous ventilation system, or a potentially faulty machine will depend on where we are and our memory of it. Others have implicated the auditory cortex, both primary and higher order [97,98], in contextual learning, albeit inferring contextual learning from its effect on tasks such as speech recognition. Subcortical structures are sensitive to the statistics of contextual sound though. Predictable, but not unpredictable, contextual signals generate more acute neural representations of sound by neurons in the inferior colliculus [99,100].

### Cortico-Subcortical Loops

A complex process such as contextual processing is likely to occur simultaneously at several interacting levels of the auditory system in a time- and task-dependent manner, possibly over progressively longer time windows [101,102]. The concept that interactions between cortical and subcortical structures might play a key role in active sensing, the flexible processing of information depending on the context and task at hand, is becoming progressively more present in the neuroscience community [100,103,104]. Corticofugal projections, which allow for cortical feedback, have been implicated in flexibility across modalities [105] and, in the auditory system, reach all the upstream levels, including the cochlear nucleus and the inferior colliculus. Indeed, in the context of noise, the cortex helps adjust the response gain to the stimulus intensity through down-regulation of the cochlear gain in loud environments, i.e., the cochlea in the case of the auditory system [106]. The inferior colliculus receives feedback projections from deep layers of the primary auditory cortex [107,108] and there is in fact substantial evidence of a role of these cortico-collicular projections in learning [109,110,111]. Indeed, inhibition of the cortico-collicular projection results in delays in learning [112] and response adaptation [96], and it prevents fast escape responses to loud sounds [113]. Understanding the role of these projections and the circuit involved will help us understand the interactions between background and foreground.

One example of such an interaction is the way in which the brain deals with the statistical structures of background noise. The intensity of background noise can vary rapidly over a wide range and auditory neurons cope by adapting their sensitivity curves to match the intensity statistics of the current context [96,114] and the extent to which neurons adapt to the statistical distribution of sound intensities increases from the auditory nerve [115] through the midbrain [114,116] to the cortex [117]. The response functions of neurons in the inferior colliculus shift to match the current distribution of sound intensities [116], and they do so with a time course suggesting that they are adapted to account for ecologically relevant sounds [118]. While we do not yet understand the time windows of integration used to detect these distributions, or even the mechanisms of this integration, we know that the dynamics of this adaptation are encoded in the inferior colliculus over relatively long time courses, inform adaptation in the cortex [118], and depend on cortico-collicular interactions [96], demonstrating the importance of listening loops in setting the cadences over which noise is assessed and dealt with by the auditory brain.

## 10. Conclusions

We have provided an overview of different perceptual and biological phenomena that demonstrate that the brain inherently expects and incorporates noise into its listening experience. It sustains mechanisms to deal with noise as a masker of signals, both to reduce its impact and to infer what lies beneath (e.g., continuity illusion). This suggests that dealing with noise is a core feature of the listening brain. Understanding how the listening brain separates signal from noise requires a combination of biological, psychophysical, and theoretical approaches and a bridge—in the form of transformative experimental design—between in vivo models and human listeners. Spanning scales of magnitude, complexity, and species, we can generate hypotheses tailored for exploration in human and animal listeners to determine how the statistics of the environmental sound, on the one hand, and the arousal state, attention, or goal-oriented behaviors, on the other, impact the ability to parse complex acoustic scenes. In awake, freely moving animals, we can establish fundamental links between brain activity and listening performance, revealing critical processes and circuits responsible for the brain’s ability to make sense of cluttered sound environments, but we are still far from understanding the listening brain’s remarkable and implicit ‘denoising’ capacity, including its ability to shape the sensitivity of the ear itself to enhance listening performance. This is necessary to establish clear links back to data and theories developed in animal models, instantiating an iterative approach to understanding the brain’s capacity for effective listening in noise.

## Data Availability

No new data were created or analyzed in this study. Data sharing is not applicable to this article.
